# Assessment and evaluation of online education and virtual simulation technology in dental education: a cross-sectional survey

**DOI:** 10.1186/s12909-024-05171-1

**Published:** 2024-02-25

**Authors:** Yu Fu, Fengqing Chu, Xiaoqing Lu, Chenxing Wang, Na Xiao, Jiajia Jiang, Jue Zheng, Hongbing Jiang

**Affiliations:** 1 Oral and Maxillofacial Surgery Medicine, Affiliated Hospital of Stomatology, Nanjing Medical University, Nanjing, China; 2 Department of Teaching Office of Stomatology, Affiliated Hospital of Stomatology, Nanjing Medical University, Nanjing, China; 3 Department of Basic Science of Stomatology, Affiliated Hospital of Stomatology, Nanjing Medical University, Nanjing, China; 4 School of Health Policy and Management, Nanjing Medical University, Nanjing, China

**Keywords:** Dental education, COVID-19, Online education, Virtual simulation education, Blended learning approach

## Abstract

**Background:**

The global outbreak of coronavirus disease (COVID-19) has led medical universities in China to conduct online teaching. This study aimed to assess the effectiveness of a blended learning approach that combines online teaching and virtual reality technology in dental education and to evaluate the acceptance of the blended learning approach among dental teachers and students.

**Methods:**

The Strengthening the Reporting of Observational Studies in Epidemiology (STROBE) checklist was followed in this study. A total of 157 students’ perspectives on online and virtual reality technology education and 54 teachers’ opinions on online teaching were collected via questionnaires. Additionally, 101 students in the 2015-year group received the traditional teaching method (TT group), while 97 students in the 2017-year group received blended learning combining online teaching and virtual reality technology (BL group). The graduation examination results of students in the two groups were compared.

**Results:**

The questionnaire results showed that most students were satisfied with the online course and the virtual simulation platform teaching, while teachers held conservative and neutral attitudes toward online teaching. Although the theoretical score of the BL group on the final exam was greater than that of the TT group, there was no significant difference between the two groups (*P* = 0.805). The skill operation score of the BL group on the final exam was significantly lower than that of the TT group (*P* = 0.004). The overall score of the BL group was lower than that of the TT group (*P* = 0.018), but the difference was not statistically significant (*P* = 0.112).

**Conclusions:**

The blended learning approach combining online teaching and virtual reality technology plays a positive role in students’ learning and is useful and effective in dental education.

## Background

Since the World Health Organization declared the global outbreak of coronavirus disease (COVID-19) in January 2020, educational institutions worldwide have shifted to online work to limit gatherings and prevent the spread of the disease [1, 2]. In particular, the education of medical students on a global scale was significantly affected. Medical education has faced enormous challenges due to the rapid spread of the pandemic [3].

Medical education is not limited to basic theoretical education but also includes operational education and clinical practice education. Since the outbreak of the pandemic, medical educators have committed to using innovative learning methods, including live lectures and virtual reality simulations, to ensure the continuity of instructional quality and motivate students to continue learning [4–6]. Video conferencing systems such as WebEx, Zoom and Tencent Meeting are used to provide online theoretical education [7]. In addition to various forms of online courses, virtual simulation technology has begun to be used to improve students’ experimental skills [8, 9].

Virtual simulation involves a computer system that can create and provide the experience of virtual worlds. The application of this technology is increasingly widespread, and in recent years, medical education has steadily begun to use virtual simulation [10–12]. Experimental teaching with medical virtual simulation is a new teaching method that relies on virtual reality, multimedia, human-computer interaction, databases and network communication to construct a highly simulated virtual experimental environment and experimental objects. An increasing number of medical schools are attempting to implement virtual simulation technology in their teaching methods to accomplish specific learning objectives and enhance more traditional teaching techniques [6].

At present, to further enhance interaction, immersion and enjoyment, the incorporation of game elements is enhancing non-game systems in various fields such as education, marketing, business administration, and medicine. The use of game elements in education is called “serious games”. Serious games refer to games that do not focus on entertainment but provide interesting virtual experiences for “gamers”, with the core purpose of learning/practicing relevant expertise [13]. The development of virtual reality technology has made serious games highly relevant in the field of medical education and health promotion. Serious games can combine entertainment technology with education and offer a positive teaching method that helps students learn [13]. Simulation teaching is another teaching strategy in the field of medical education that can provide a relatively safe practice environment for learners and patients [14]. Simulation teaching has the potential to help learners acquire technical skills and nontechnical skills, including leadership, teamwork, and communication. While there are some differences between serious games and simulation teaching, many serious games have implemented simulation and virtual reality technologies. These games involve asynchronous learning where students can improve their skills in a safe environment with a “simulation-based concept.” Currently, simulation-based serious games, such as the Virtual Education System for Dentistry, iDental, and PerioSim, are widely applied in dental education [15].

Before the COVID-19 era, online teaching was not the most common teaching method used in medical education in China. Nevertheless, some medical schools are becoming ready to switch from conventional teaching methods to virtual classrooms [12, 13]. Online teaching practices during the epidemic have provided a research foundation for the transformation of teaching modes. This study aimed to evaluate students’ and teachers’ perceptions of online theoretical teaching and virtual simulation teaching during the epidemic through a questionnaire survey and to compare the difference between the blended learning approach combining online teaching and virtual reality technology and the traditional teaching model based on graduation examination scores. The effects of this teaching strategy on student learning outcomes were examined to provide a fresh viewpoint for selecting and developing educational models in the future.

## Methods

### Participants

A total of 203 students admitted to Nanjing Medical University School of Stomatology in 2017 and 2018 and 70 teachers were included in our questionnaire study. A total of 198 students admitted in 2015 and 2017 were included in the achievement comparison study, including 101 students admitted in 2015 (2015-year group) and 97 students admitted in 2017 (2017-year group). The students were grouped according to their grades. Students admitted in 2015 received traditional teaching methods (Group TT) and hybrid teaching methods (online instructions paired with a virtual simulation operating platform) were used, as were the students admitted in 2017 (Group BL). The inclusion criteria for admission to the study were as follows: (1) voluntary participation in this study and (2) completion of the whole semester. The teachers and students who did not voluntarily participate in the study or withdrew from the study for various reasons were excluded. The students, who were all older than 16 years of age, provided informed consent to voluntarily participate in the study. All the responses remained anonymous throughout the study. All methods were performed in accordance with the Declaration of Helsinki.

### Online education

Synchronous teaching was conducted through live video software (Tencent Meeting, Ding Talk, etc.) with the addition of online discussion sessions to instantly address students’ queries. Asynchronous teaching was performed through a teaching platform (Superstar Learning Platform, Rain Classroom, etc.) before and after class. Before class, teachers distributed tasks to help students to study independently. After class, the students could use software replay for review or watch recorded lessons for further learning.

### Virtual simulation teaching

**Fig. 1:**
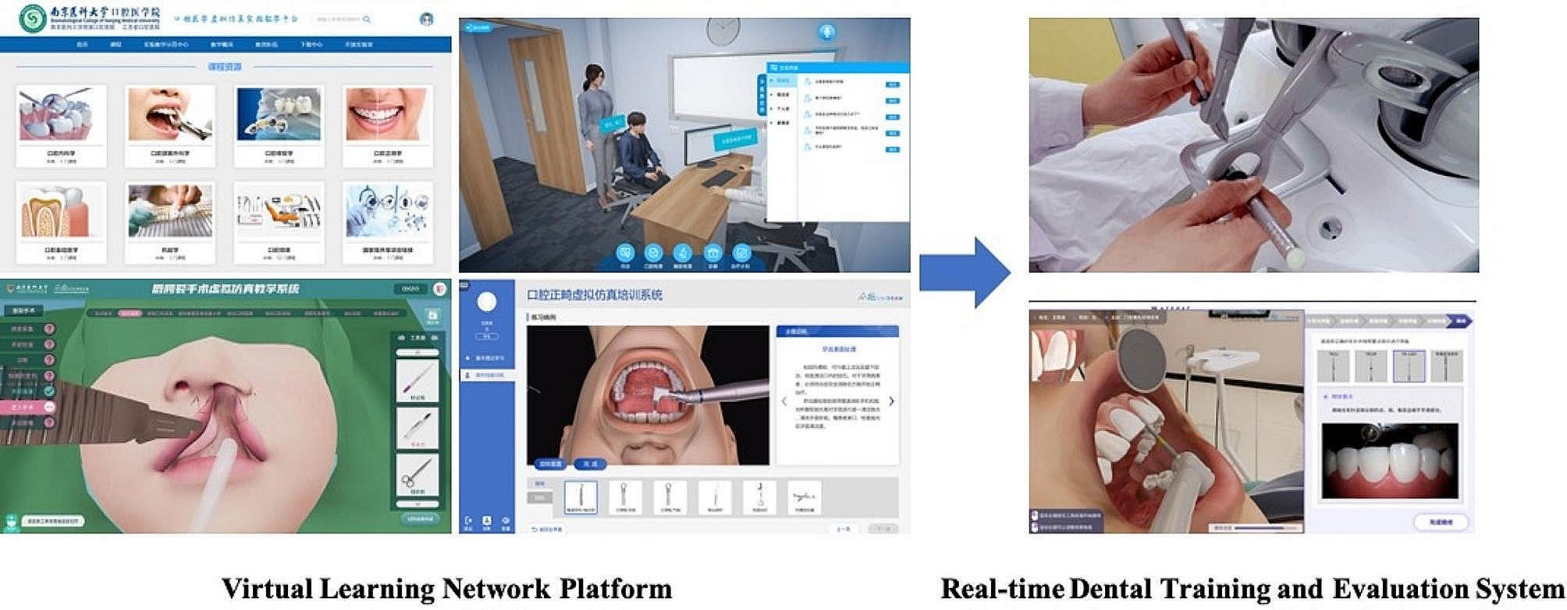
Flowchart showing the use of the virtual operating platform

The Virtual Education System for Dentistry, which includes the Virtual Learning Network Platform (VLNP) and Real-time Dental Training and Evaluation System (RDTES), was used for virtual simulation teaching. Students registered for the VLNP for online simulation-based serious games and assessment under the premise of learning fundamental theoretical concepts through standardized videos and online courses. After passing the assessment, students could schedule an appointment to enter the open laboratory and use the RDTES surgery simulator. Three-dimensional virtual and force rendering technology was used in the oral virtual simulation surgical simulator (Fig. 1). There were several force tactile feedback virtual simulation experimental projects, such as “straight arch fixed orthodontic appliance bonding”, “clinical thinking teaching and examination system”, and “tooth extraction”. These projects involved realistic simulation of clinical treatment and the operating environment, training students to think about clinical treatment, and standardizing students’ clinical operation skills.

### Sample size

The single proportion of the population formula [n = Zα^2^.p(1-p)/d^2^] was used to determine the minimum sample size. The maximum sample size was guaranteed determined for a 95% confidence interval (Zα = 1.96), 5% margin of error and 50% population proportion. A total of 310 students and 73 teachers were included. For possible incomplete or nonresponding questionnaires, we increased the calculated sample size by 10%, resulting in final sample sizes of 203 students and 70 teachers.

### Questionnaire survey

The questionnaire used in this study was developed based on a review of the relevant literature and input from faculty and students. The questionnaire concerning online classrooms included 12 items for students and 10 items for teachers; these items were used to summarize the benefits and potential drawbacks of conducting classes online. The students’ questions 1–7 were categorized as benefits, whereas questions 8–12 were categorized as drawbacks. The teachers’ questions 1–5 related to advantages, whereas questions 6–10 related to drawbacks. The questionnaire on the virtual operating platform consisted of 10 entries that summarized information on the usability and effectiveness of the virtual operating platform. Questions 1–4 corresponded to usability, and questions 6–10 corresponded to effectiveness. The purpose of this survey was (1) to assess the advantages and disadvantages of online classes, (2) to assess the convenience of using the virtual operating platform, (3) to reflect the teaching effectiveness of the virtual operating platform, and (4) to assess the acceptance of the virtual operating platform and online classes. There were five alternatives on a Likert scale, from strongly agree to strongly disagree, for the responses to the questions. The data were collected using an online platform named WJX (https://www.wjx.cn), which provides functions equivalent to Amazon Mechanical Turk. A detailed description of the purpose of the study, the study team, confidentiality rights, and withdrawal from the study was included in the cover letter of the questionnaire. The purpose of the study was explained to the participants by the investigator, emphasizing that their personal information and responses would be treated with confidentiality. Additionally, participants were instructed not to engage in discussions regarding the questionnaire with other participants. All participants provided consent before starting to answer the questionnaire. To avoid information and memory bias, participants were asked to complete the questionnaire anonymously after the course was completed. Strict quality-control measures were implemented to ensure the quality of the data. Each participant could submit survey responses only once, and no edits could be made after submission.

### Comparison of the teaching effects between group TT and group BL

The graduation examination scores of dental students in Group TT and Group BL were selected for analysis. The graduation examination included two parts: theory examination and skill operation examination. The skill operation was conducted according to the procedure of the dental qualification examination. The same examiners were trained to score and evaluate the results with reference to the same scoring standard.

### Outcome measurements

#### Questionnaires

To ensure the quality of the questionnaires, three dental education experts evaluated the validity of the questionnaire content and repeatedly revised the questions until the objective consistency index of each item was greater than 0.5. In terms of reliability, Items in question were modified or removed until all structures had a Cronbach’s alpha coefficient above 0.8. The Cronbach’s alpha for internal consistency and reliability was 0.875. Before using the questionnaires, pilot study was conducted with 10 teachers and 20 students to evaluate the clarity, feasibility and practicability of the questionnaires, and to make modifications. The teachers and students who participated in the pilot study were not included in the study sample.

#### Examination assessments

The content of the examination was evaluated by three dental education experts who used content validity to confirm its suitability for achieving the expected learning outcomes. The examination was piloted and evaluated considering internal consistency. The examination was modified until Cronbach’s alpha coefficient was above 0.7.

### Statistical analysis

The independent-sample t test was used to compare the differences in the results between the two groups. All the data are expressed as the means ± standard deviations (means ± SDs), and a significant difference test was performed using a significance level of 0.05. All the statistical analyses were performed with SPSS version 24.0.

## Results

### Survey results

A total of 157 students completed the questionnaire, for an effective recovery rate of 77.34%. The study sample was composed of 73 (46.50%) students admitted in 2018 (*n* = 73) and 84 (53.50%) students admitted in 2017. A total of 54 teachers completed the questionnaire, for an effective recovery rate of 77.14%. Table 1 lists demographic information of teachers. The participating teachers all possessed over five years of teaching experience, had at least a master’s degree, and the majority of the them demonstrate proficiency in computer utilization.

**Table 1:** Participant characteristics (*N* = 54)

Variables	Numbers (*N*)	Percent (%)
**Gender**		
Male	23	42.6
Female	31	57.4
**Age (years)**		
25–34	25	46.3
35–44	18	33.3
≥ 45	11	20.4
**Professional title**		
Intermediate title	33	61.1
Senior title	21	38.9
**Teaching years**		
6–10 years	42	77.8
> 10 years	12	22.2
**Self-evaluation of teaching ability**		
Excellent	10	18.5
Ordinary	37	68.5
Poor	7	13
**Computer skill**		
Proficient	37	68.5
General	15	27.8
Primary	2	3.7

### Students’ attitudes toward online education

**Fig. 2:**
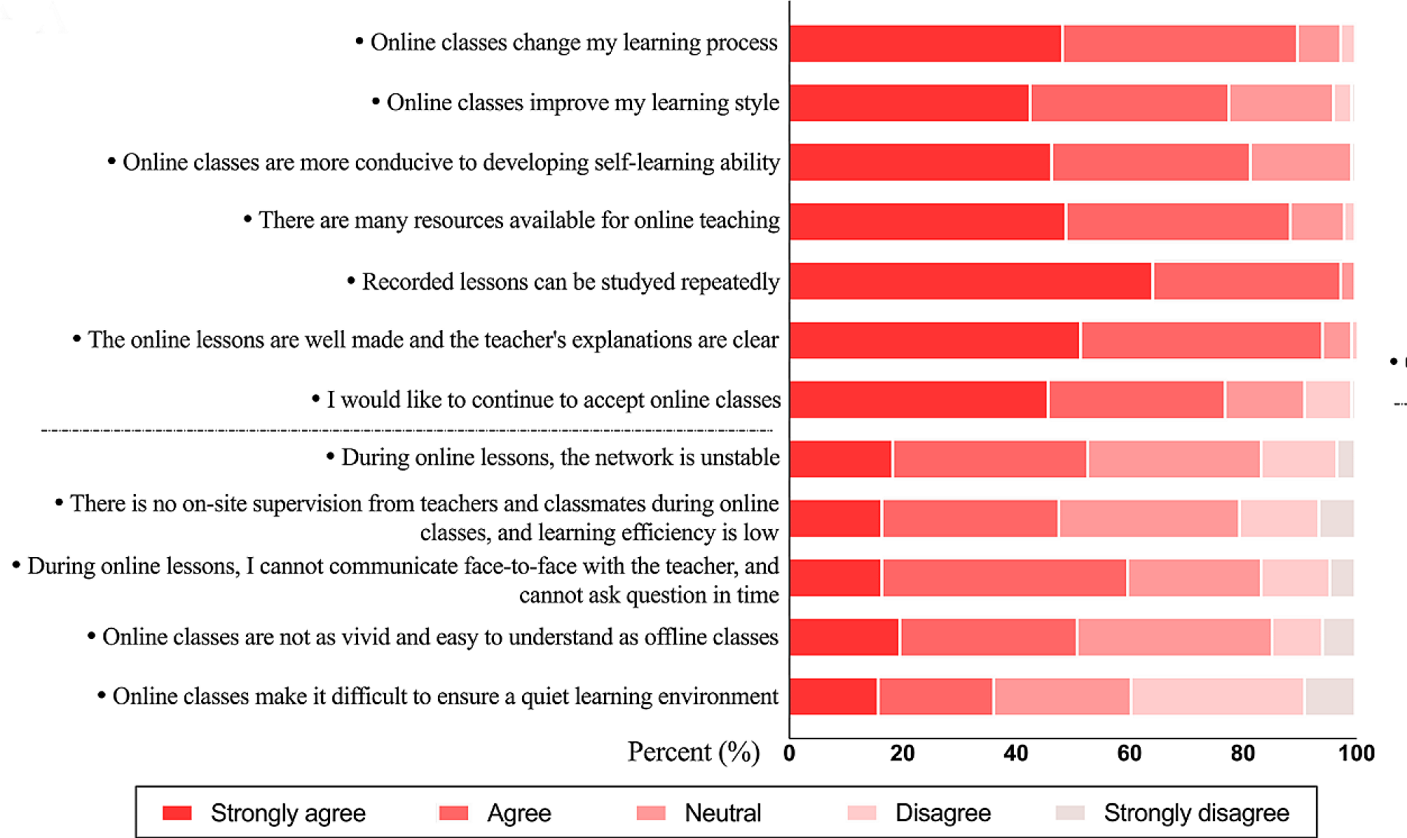
Questionnaire results of students’ responses to online teaching

The Likert responses to each question on the questionnaire about students’ perceptions of online teaching are shown in Fig. 2. Nearly half of the students strongly agreed that online education changed their learning process (48.41%) and learning style (42.68%). A total of 46.55% thought that online classes were better at developing their independent learning skills, and 49.04% of the students thought that there were more resources available for online learning. A high percentage of the students (64.33%) felt that online recorded classes could be watched repeatedly. More than half of the students (51.59%) thought that the online classes were well produced and that the teachers’ explanations were clear. A total of 45.86% of the students were willing to continue to receive online teaching. Regarding the possible disadvantages of online lessons, 34.39% of the students thought that the network was unstable and latent when taking online lessons, while 30.57% of students were neutral about this disadvantage. A total of 31.85% of the students were neutral about the lack of on-site supervision by teachers and classmates and the low learning efficiency of online lessons, while 31.21% of students agreed with this opinion. A total of 43.31% of the students agreed that they could not communicate face-to-face with the teacher during online classes and could not obtain answers to points they did not understand in a timely manner. Furthermore, 34.39% of the students were neutral to the idea that online classes were not as lively and easy to understand as offline classes were, while 31.21% of the students agreed with this idea. A total of 30.57% of the students did not feel that online learning made it difficult to ensure a quiet learning environment.

### Teachers’ attitudes toward online education

**Fig. 3:**
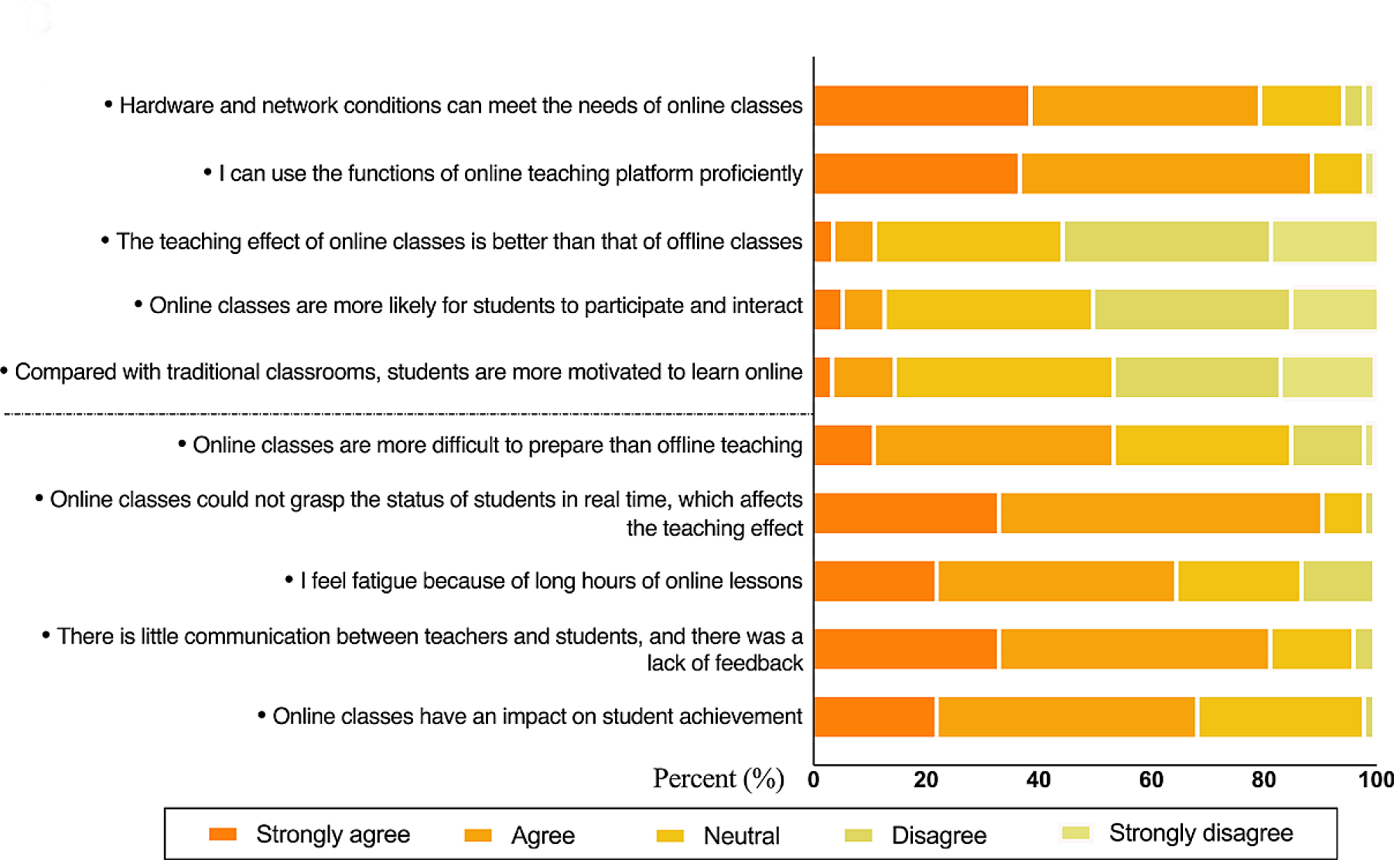
Questionnaire results of teachers’ responses to online teaching

The Likert responses to the questionnaire for each of the teachers’ questions about online teaching are shown in Fig. 3. Most teachers believed that hardware and network conditions could meet the needs of online classes (79.63%) and that they were proficient in using online teaching platforms (88.89%). A total of 55.56% of the teachers did not think that online teaching was better than offline teaching was, 18.52% strongly disagreed with this view, and 33.33% (18/54) of the teachers had a neutral attitude. Among the teachers, 37.04% were neutral to the view that online students were more able to participate in interaction, while half believed that online classes did not improve students’ participation. A total of 38.89% of the teachers believed that students did not necessarily have stronger learning motivation for online classes than traditional teaching, and 46.30% of the teachers believed that students lacked learning motivation for online classes. Additionally, 72.22% of the teachers believed that online class preparation was more difficult than offline class preparation was, while 31.48% of the teachers had a neutral attitude. Furthermore, 90.74% of the teachers felt that online classes could not support students in real time, which influenced teaching effectiveness. A total of 64.81% of the teachers thought that long hours of online teaching made them feel tired, while 22.22% of the teachers had a neutral attitude. Among the teachers, 81.48% felt that there was insufficient communication and feedback between teachers and students after class, 68.52% believed that online classes had an impact on student performance, and 29.63% thought that online classes did not necessarily have an effect on student performance.

### Students’ attitudes toward the virtual operating platform

**Fig. 4:**
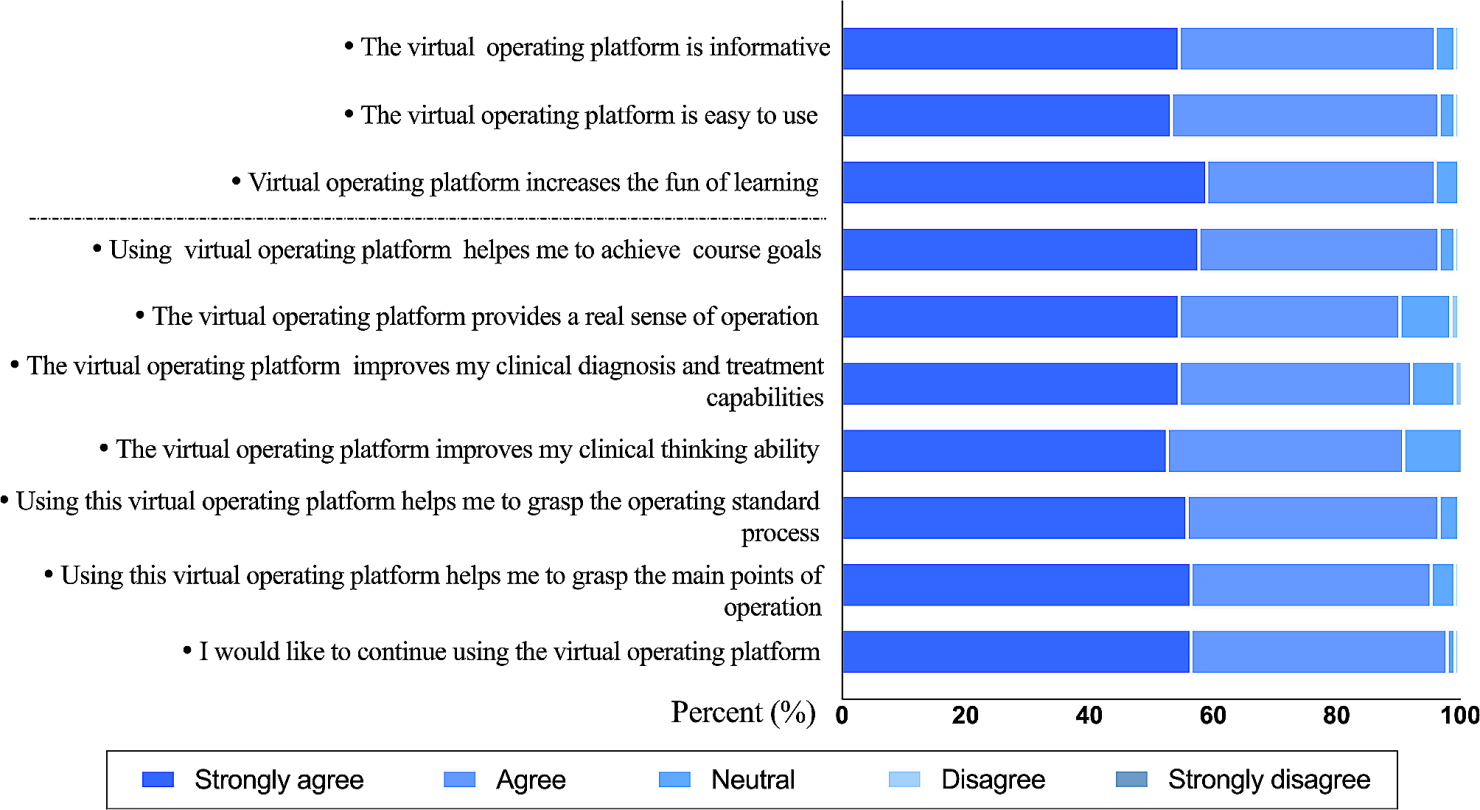
Questionnaire results of students’ responses to the virtual operating platform

The Likert responses to the questionnaire on the students’ questions about the virtual operating platform are shown in Fig. 4. More than half of the students strongly agreed that the virtual operating platform was rich in content (54.78%) and easy to use (53.5%). A total of 59.24% of the students felt that the virtual operating platform could increase their enjoyment of learning, and 57.96% of the students thought that using the virtual operating platform was more conducive to achieving course objectives. A total of 54.78% of the students thought that the virtual simulation operating platform provided a sense of real operation, more than half of the students strongly agreed that the virtual operating platform helped them improve their clinical diagnosis and treatment skills (54.78%), and believed that the virtual operating platform helped to improve their thinking ability for clinical diagnosis and treatment (52.87%). Approximately 56% of the students felt that the virtual operating platform made it easier for them to master the standard operating process and the operating points. Finally, 56.69% of the students were willing to continue to use the virtual operating platform.

### Results of scoring

**Fig. 5:**
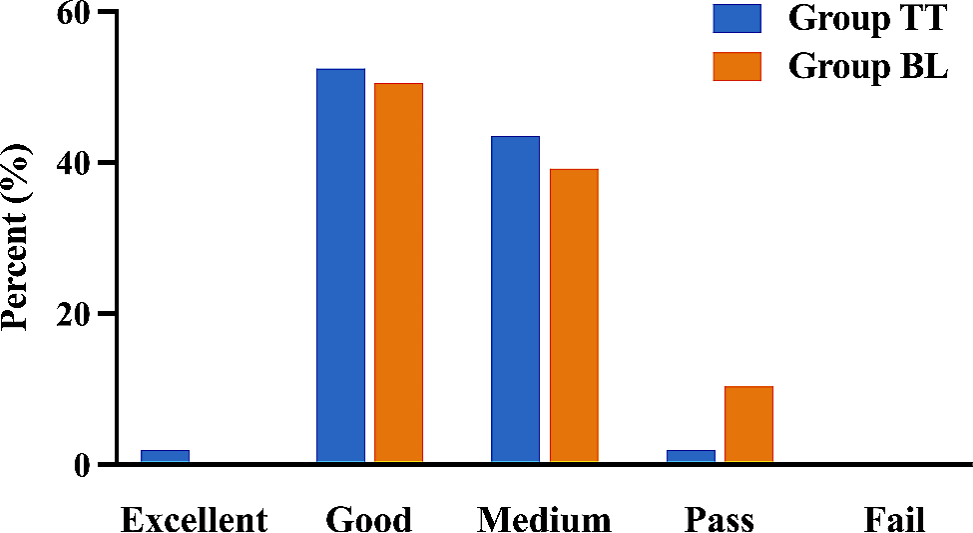
Comparison of the graduation examination scores between the TT and BL groups. Excellent: score > 90; Good: score ranging from 80 to 90; Medium: score ranging from 70 to 80; Pass: score ranging from 60 to 70; Fail: score < 60

A comparison of student performance between the blended teaching method of online teaching combined with virtual simulation teaching and the traditional teaching method is shown in Table 2. The average theory exam score of Group TT was 71.94 (SD = 10.037), which was lower than the score for Group BL (mean = 72.23, SD = 6.381, *P* = 0.805). The average score on the operating skill exam in Group BL was 85.38 (SD = 8.437), which was lower than the score in Group TT (mean = 88.23, SD = 5.225; *P* = 0.004). The total score of the students’ graduation examination results in Group BL was 78.81 (SD = 6.008), while the score in Group TT was 80.08 (SD = 5.282). Moreover, there was no significant difference between the two groups (*P* = 0.112). Segmental analysis of the total scores showed that the number of excellent students was greater in Group TT than in Group BL, the number of good and medium students was similar in both groups, and the number of passing students was lower in Group TT than in Group BL (Fig. 5).

**Table 2:** The scores of the graduation examination

Scoring results	Group TT (*n* = 101)	Group BL(*n* = 97)	*T* value	*P* value
Theory exam	71.94 ± 10.037	72.23 ± 6.381	-0.246	0.805
Skill operation exam	88.23 ± 5.225	85.38 ± 8.437	2.875	**0.004** ^*^
Total score	80.08 ± 5.282	78.81 ± 6.008	1.593	0.112

^*^P < 0.05

## Discussion

Under the impact of the epidemic, Chinese medical schools have encouraged teachers to change teaching methods and conduct online teaching of theory courses to provide students with distance learning opportunities to minimize the impact of the epidemic on medical students’ learning. The Nanjing Medical University Stomatology School actively explored new teaching methods and developed a blended learning approach that combines online and virtual reality technology to ensure the quality of teaching for dentistry students. By administering questionnaires and comparing scores, we explored the teaching effect of the new teaching approach. We propose suggestions and opinions for future teaching reform.

Due to the impact of the epidemic, many digital teaching concepts and research findings have emerged in the field of medical teaching worldwide [16, 17]. Abbasi S et al. concluded that most students have a negative view of online learning, which may be related to internet familiarity and usage [18]. Cheng et al. showed that despite the impact of online learning as a new way of studying students’ learning styles and progress, most students still accept online learning [16]. This study revealed that students were more receptive to online courses. Most students believed that online courses were more conducive to developing students’ independent learning skills by changing their learning style. Nevertheless, some students felt that online courses were less efficient because they were not supervised by teachers on site, while other students were neutral about this viewpoint. This may be related to students’ study habits. For students with good self-discipline, the effect on learning efficiency of whether the course was supervised by a teacher or classmates was not significant.

The use of an e-learning platform to teach theoretical content can be divided into two forms, synchronous teaching and asynchronous teaching, according to whether teachers and students are online at the same time [19, 20]. Synchronous teaching mainly refers to online live teaching, which can achieve synchronous interaction in the teaching process. Teachers and students can interact in real time through questions and connections, and students can directly provide feedback on difficult knowledge points [21]. Asynchronous teaching is conducted through interactive teaching methods such as video, which is flexible in time and space. Teachers upload teaching resources to the teaching platform in advance, and students can arrange time to learn independently [22, 23]. Previous studies have shown that students are more interested in asynchronous learning, although it involves less teacher‒student interaction [24]. Our research showed that in asynchronous teaching classes, students could watch knowledge points repeatedly, which was conducive to consolidating the knowledge points and providing students with a pleasant learning experience through efficient and diverse extended learning. However, asynchronous teaching courses affect effective interactions between teachers and students, and most students believe that they cannot communicate with teachers face-to-face in online courses and that they cannot receive timely answers to their questions. The synchronous teaching course was convenient for real-time communication between teachers and students, but the live course was strongly affected by objective factors such as network speed and software, and some students believed that there were unstable networks and connection lags in online courses. The integration of synchronous teaching and asynchronous teaching can provide complementary advantages and improve the effectiveness of online teaching.

Online teaching is also a challenge for teachers [25]. It is difficult to transform teachers’ teaching concepts from traditional teaching to online teaching. Teaching spaces, teacher preparation methods, teaching methods, and teacher‒student interactions all need to be adjusted. Although the online network platform is rich in variety and relatively simple to operate, for some teachers, the operation of online teaching equipment and the use of the network platform affect the quality of teaching. It is difficult for teachers to optimize students’ learning using corresponding teaching software, which may further reduce students’ enthusiasm and initiative for learning. In this study, teachers’ attitudes toward online teaching were somewhat negative. Although the hardware and software conditions met the needs of online courses and teachers could use online teaching platforms proficiently, they believed that the teaching effect of online teaching was not necessarily better than that of offline classes. This might be related to factors such as the inability to support students in real time and the lack of communication between teachers and students. In addition, online class preparation was difficult, and long-term online teaching made teachers feel tired, which might also influence the teaching effect. The difference in the attitudes of teachers and students toward online classes might be related to the transformation of teachers’ teaching methods and concepts.

Dental education has its own characteristics and contains many practice-based courses, but online education cannot provide clinical teaching and practice experience [26]. Digital and multimedia teaching is gradually becoming a future trend to supplement classroom presentations and laboratory teaching. In recent years, digital interactive systems have played an important and unique role in the process of medical education [27, 28]. The virtual simulation teaching system has gradually been applied in various branches of discipline. Virtual simulation with three-dimensional software technology has been used to construct the human body model so that students can understand the anatomical structure more intuitively through a “roaming” interactive mode, not only to stimulate students’ interest in learning but also to concretize and visualize abstract content, which can improve student interaction [29–31]. In addition, through simulation, students’ basic knowledge can be consolidated more effectively. Carlos M. Serrano et al. assessed the educational implementation of virtual reality and haptics as well as school satisfaction and reported that most schools were satisfied with virtual reality haptic dental trainers [32]. In the past three years, our college has designed and developed virtual simulation teaching platforms for oral anatomy and physiology, endodontics, oral and maxillofacial surgery, prosthodontics and orthodontics and applied them in teaching practice during the epidemic. The results of the questionnaire showed that students were satisfied with the virtual simulation platform in terms of ease of use, effectiveness, and content abundance. Almost all of the students’ comments were positive. Most students agreed that the virtual operating platform could help them grasp the standard procedure and master the key points of operation and could effectively improve clinical diagnosis and thinking skills. The results indicated that the virtual operating platform had certain advantages and was suitable for continued application in teaching practical skills.

Blended learning is a mixture of traditional face-to-face learning and simultaneous or asynchronous online learning. This approach is regarded as a promising substitute for medical education because of its integrative nature [33]. Furthermore, this method has been reported to improve the clinical practice of medical students [34]. However, studies of blended teaching methods for online education and virtual simulation technology have rarely been reported. We compared and analyzed the student test scores for two grades to determine the teaching efficacy of the blended teaching method, which combined online learning with virtual simulation training. There was no significant distinction between the scores of students who received blended teaching and those who received traditional teaching for theory classes, suggesting that online education had an impact on learning that was comparable to that of offline education and that online education did not affect students’ learning outcomes.

In recent years, an increasing number of researchers have focused on empirical research on the role of virtual simulation or simulation-based serious games in improving the effectiveness of dental education [14]. Rasa Mladenovic et al. found that in the age of COVID-19, serious games involving local anesthesia can improve students’ knowledge and skills as an additional e-learning tool [35]. Lemos et al. found that serious play can lead to positive perceptions, can be used to motivate students to learn, and can achieve better learning outcomes than traditional learning [36]. In our study, the teaching effect of using the virtual simulation platform was marginally inferior to that of traditional offline skills training in terms of practical skills, which was potentially attributed to incomplete content coverage in the early stages of platform development and students’ unfamiliarity with the system. The virtual simulation platform can therefore be used in addition to traditional skills training but cannot replace offline skills training. Notably, previous studies have focused on the impact of one type of simulation on learning outcomes. In our study, our Virtual Education System for Dentistry consisted of two parts: a serious simulation-based game and a surgical simulator with a feedback system. The content of the simulation included clinical diagnosis and treatment simulation and clinical skill operation. According to Bloom’s taxonomy, there are six progressive levels of learning from the foundation to the top in the following order: knowledge, understanding, application, analysis, synthesis, and evaluation. Through the utilization of our Virtual Education System for Dentistry, we anticipate improvements in students’ clinical reasoning and operational proficiency through advanced training. Further research is being conducted on the application of the Virtual Education System for Dentistry.

Overall student performance did not differ significantly between the two teaching approaches, demonstrating that objective factors such as epidemic-related school closures had no impact on how effectively pupils learned. The blended teaching approach was able to offer a superior teaching outcome during the pandemic and played a significant role in its application. Considering the advantages of blended teaching methods, further research can be conducted on teaching practices promoted and applied in medical schools. In Kirkpatrick’s model, the evaluation system is divided into four levels: how participants respond to the project, what they can learn from the project, whether and how the project changes their practice behavior, and the far-reaching impact of the project on participants. In this regard, further research should focus on assessing the far-reaching impact of blended teaching models on students, similar to the third and fourth levels of Kirkpatrick’s model.

The generalizability theory is used to accurately estimate the reliability of complex tests that involve multiple sources of measurement error, including generalizability studies (G studies) and decision studies (D studies) [37]. The generalizability theory is suitable for the reliability analysis of tests involving many influencing factors, such as clinical skills. Most analyses included participant, evaluator, evaluator, and interaction as factors. At present, generalizability theory has become the mainstream theory of quality analysis for practical ability evaluation, and a plethora of studies have applied it to OSCE [38]. In our study, Cronbach’s coefficient was used to measure and evaluate the examination results. In future studies, generalizability theory should be considered to comprehensively evaluate the influence of various factors on students’ scores, further improve the test design and optimize the reliability of the test.

### Limitations

There were several limitations to our study, mainly in two aspects. On the one hand, there were limitations in the use of learning tools. Currently, no artificial intelligence teaching method can simulate real patient and diagnosis and treatment processes, and more informative projects need to be developed. The virtual simulation platform lacked mechanical feedback, and the current use of offline simulators with force feedback was limited by development costs and could not be promoted on a large scale. Additionally, the subjects of this study were fourth-year university students, but internship students were excluded. The teaching effect of virtual simulation on students in the internship stage needs further research. It may be more appropriate to compare learner performance among students in the same cohort because different effects come from different learning strategies, different groups of learners, or the difficulty level of tests.

## Conclusions

The results of this study showed that students were more satisfied with the combination of online teaching and virtual simulation teaching than with traditional teaching. However, compared with traditional teaching methods, the application of a blended teaching mode had no significant impact on the overall performance of students. Based on the advantages of the blended teaching method in our study, this approach has high potential for application.

## Data Availability

All data generated or analyzed during this study are included in this published article.
